# Encoding Individual Source Sequences for the Wiretap Channel

**DOI:** 10.3390/e23121694

**Published:** 2021-12-17

**Authors:** Neri Merhav

**Affiliations:** The Viterbi Faculty of Electrical and Computer Engineering, Technion-Israel Institute of Technology, Technion City, Haifa 3200003, Israel; merhav@technion.ac.il; Tel.: +972-4-8294737

**Keywords:** wiretap channel, physical layer security, semantic security, individual sequence, finite-state machine, Lempel–Ziv algorithm, side information

## Abstract

We consider the problem of encoding a deterministic source sequence (i.e., individual sequence) for the degraded wiretap channel by means of an encoder and decoder that can both be implemented as finite-state machines. Our first main result is a necessary condition for both reliable and secure transmission in terms of the given source sequence, the bandwidth expansion factor, the secrecy capacity, the number of states of the encoder and the number of states of the decoder. Equivalently, this necessary condition can be presented as a converse bound (i.e., a lower bound) on the smallest achievable bandwidth expansion factor. The bound is asymptotically achievable by Lempel–Ziv compression followed by good channel coding for the wiretap channel. Given that the lower bound is saturated, we also derive a lower bound on the minimum necessary rate of purely random bits needed for local randomness at the encoder in order to meet the security constraint. This bound too is achieved by the same achievability scheme. Finally, we extend the main results to the case where the legitimate decoder has access to a side information sequence, which is another individual sequence that may be related to the source sequence, and a noisy version of the side information sequence leaks to the wiretapper.

## 1. Introduction

In his seminal paper, Wyner [1] introduced the wiretap channel as a model of secure communication over a degraded broadcast channel, without using a secret key, where the legitimate receiver has access to the output of the good channel and the wiretapper receives the output of the bad channel. The main idea is that the excess noise at the output of the wiretapper channel is utilized to secure the message intended to the legitimate receiver. Wyner fully characterized the best achievable trade-off between reliable communication to the legitimate receiver and the equivocation rate at the wiretapper, which was quantified in terms of the conditional entropy of the source given the output of the wiretapper channel. One of the most important concepts, introduced by Wyner, was the *secrecy capacity*, that is, the supremum of all coding rates that allow both reliable decoding at the legitimate receiver and full secrecy, where the equivocation rate saturates at the (unconditional) entropy rate of the source, or equivalently, the normalized mutual information between the source and the wiretap channel output is vanishingly small for large blocklength. The idea behind the construction of a good code for the wiretap channel is basically the same as the idea of binning: one designs a big code, that can be reliably decoded at the legitimate receiver, which is subdivided into smaller codes that are fed by purely random bits that are unrelated to the secret message. Each such sub-code can be reliably decoded individually by the wiretapper to its full capacity, thus leaving no further decoding capability for the remaining bits, which all belong to the real secret message.

During the nearly five decades that have passed since [1] was published, the wiretap channel model was extended and further developed in many aspects. We mention here just a few. Three years after Wyner, Csiszár and Körner [2] extended the wiretap channel to a general broadcast channel that is not necessarily degraded, allowing also a common message intended to both receivers. In the same year, Leung-Yan-Cheong and Hellman [3], studied the Gaussian wiretap channel, and proved, among other things, that its secrecy capacity is equal to the difference between the capacity of the legitimate channel and that of the wiretap channel. In [4], Ozarow and Wyner considered a somewhat different model, known as the type II wiretap channel, where the channel to the legitimate receiver is clean (noiseless), and the wiretapper can access a subset of the coded bits. In [5], Yamamoto extended the wiretap channel to include two parallel broadcast channels that connect one encoder and one legitimate decoder, and both channels are wiretapped by wiretappers that do not cooperate with each other. A few years later, the same author [6] further developed the scope of [1] in two ways: first, by allowing a private secret key to be shared between the encoder and the legitimate receiver, and secondly, by allowing a given distortion in the reproducing the source at the legitimate receiver. The main coding theorem of [6] suggests a three-fold separation principle, which asserts that no asymptotic optimality is lost if the encoder first applies a good lossy source code, then encrypts the compressed bits, and finally, applies a good channel code for the wiretap channel. In [7], this model in turn was generalized to allow source side information at the decoder and at the wiretapper in a degraded structure with application to systematic coding for the wiretap channel. The Gaussian wiretap channel model of [3] was also extended in two ways: the first is the Gaussian multiple access wiretap channel of [8], and the second is Gaussian interference wiretap channel of [9,10], where the encoder has access to the interference signal as side information. Wiretap channels with feedback were considered in [11], where it was shown that feedback is best used for the purpose of sharing a secret key as in [6,7]. More recent research efforts were dedicated to strengthening the secrecy metric from weak secrecy to strong secrecy, where the mutual information between the source and the wiretap channel output vanishes, even without normalization by the blocklength, as well as to semantic security, which is similar but refers even to the worst-case message source distribution; see, for example, Refs. [12,13,14,15,16], (Section 3.3 in [14]).

In this work, we look at Wyner’s wiretap channel model from a different perspective. Following the individual sequence approach pioneered by Ziv in [17,18,19], and continued in later works, such as [20,21], we consider the problem of encoding a deterministic source sequence (i.e., an individual sequence) for the degraded wiretap channel using finite-state encoders and finite-state decoders. One of the non-trivial issues associated with individual sequences, in the context of the wiretap channel, is how to define the security metric, as there is no probability distribution assigned to the source, and therefore, the equivocation, or the mutual information between the source and the wiretap channel output, cannot be well defined. In [20], a similar dilemma was encountered in the context of private key encryption of individual sequences, and in the converse theorem therein, it was assumed that the system is perfectly secure in the sense that the probability distribution of the cryptogram does not depend on the source sequence. In principle, it is possible to apply the same approach here, where the word ‘cryptogram’ is replaced by the ‘wiretap channel output’. However, in order to handle residual dependencies, which will always exist, it would be better to use a security metric that quantifies those small dependencies. To this end, it makes sense to adopt the above-mentioned maximum mutual information security metric (or, equivalently the semantic security metric), where the maximum is over all input assignments. After this maximization, this quantity depends only on the ‘channel’ between the source and the wiretap channel output.

Our first main result is a necessary condition (i.e., a converse to a coding theorem) for both reliable and secure transmission, which depends on: (i) the given individual source sequence, (ii) the bandwidth expansion factor, (iii) the secrecy capacity, (iv) the number of states of the encoder, (v) the number of states of the decoder, (vi) the allowed bit error probability at the legitimate decoder and (vii) the allowed maximum mutual information secrecy. Equivalently, this necessary condition can be presented as a converse bound (i.e., a lower bound) to the smallest achievable bandwidth expansion factor. The bound is asymptotically achievable by Lempel–Ziv (LZ) compression followed by a good channel coding scheme for the wiretap channel. Given that this lower bound is saturated, we then derive also a lower bound on the minimum necessary rate of purely random bits needed for adequate local randomness at the encoder, in order to meet the security constraint. This bound too is achieved by the same achievability scheme, a fact which may be of independent interest regardless of individual sequences and finite-state encoders and decoders (i.e., also for ordinary block codes in the traditional probabilistic setting). Finally, we extend the main results to the case where the legitimate decoder has access to a side information sequence, which is another individual sequence that may be related to the source sequence, and where a noisy version of the side information sequence leaks to the wiretapper. It turns out that in this case, the best strategy is the same as if one assumes that the wiretapper sees the clean side information sequence. While this may not be surprising as far as sufficiency is concerned (i.e., as an achievability result), it is less obvious in the context of necessity (i.e., a converse theorem).

The remaining part of this article is organized as follows. In Section 2, we establish the notation, provide some definitions and formalize the problem setting. In Section 3, we provide the main results of this article and discuss them in detail. In Section 4, the extension that incorporates side information is presented. Finally, in Section 5, the proofs of the main theorems are given.

## 2. Notation, Definitions, and Problem Setting

### 2.1. Notation

Throughout this paper, random variables are denoted by capital letters; specific values they may take are denoted by the corresponding lower case letters; and their alphabets are denoted by calligraphic letters. Random vectors, their realizations, and their alphabets are denoted, respectively, by capital letters, the corresponding lower case letters and calligraphic letters, all superscripted by their dimensions. For example, the random vector $X^{n}=\left(X_{1},\ldots ,X_{n}\right)$, (*n* – positive integer) may take a specific vector value $x^{n}=\left(x_{1},\ldots ,x_{n}\right)$ in $X^{n}$, the *n*-th order Cartesian power of X, which is the alphabet of each component of this vector. Infinite sequences are denoted using the bold face font, e.g., $x=(x_{1},x_{2},\ldots )$. Segments of vectors are denoted by subscripts and superscripts that correspond to the start and the end locations; for example, $x_{i}^{j}$, for i<j integers, denotes $\left(x_{i},x_{i+1},\ldots ,x_{j}\right)$. When i=1, the subscript is omitted.

Sources and channels are denoted by the letter *P* or *Q*, subscripted by the names of the relevant random variables/vectors and their conditionings, if applicable, following the standard notation conventions, e.g., $Q_{X}$, $P_{Y|X}$, and so on, or by abbreviated names that describe their functionality. When there is no room for ambiguity, these subscripts are omitted. The probability of an event E will be denoted by Pr{E}, and the expectation operator with respect to (w.r.t.) a probability distribution *P* is denoted by $E_{P}\left\{\cdot \right\}$. Again, the subscript is omitted if the underlying probability distribution is clear from the context or explicitly explained in the following text. The indicator function of an event E is denoted by 1{E}, that is, 1{E}=1 if E occurs; otherwise, 1{E}=0.

Throughout considerably large parts of the paper, the analysis is carried out w.r.t. joint distributions that involve several random variables. Some of these random variables are induced from empirical distributions of deterministic sequences, while others are ordinary random variables. Random variables from the former kind are denoted with ‘hats’. As a simple example, consider a deterministic sequence, $x^{n}$, that is fed as an input to a memoryless channel defined by a single-letter transition matrix, $\left\{P_{Y|X},\;x\in X,\;y\in Y\right\}$, and let $y^{n}$ denote a realization of the corresponding channel output. Let $P_{\hat{X}\hat{Y}}\left(x,y\right)=\frac{1}{n}\sum_{i=1}^{n}1\left\{x_{i}=x,\;y_{i}=y\right\}$ denote the joint empirical distribution induced from $\left(x^{n},y^{n}\right)$. In addition to $P_{\hat{X}\hat{Y}}\left(x,y\right)$, we also define $P_{\hat{X}Y}\left(x,y\right)=E\left\{P_{\hat{X}\hat{Y}}\left(x,y\right)\right\}$, where now *Y* is an ordinary random variable. Clearly, the relation between the two distributions is given by $P_{\hat{X}Y}\left(x,y\right)=P_{\hat{X}}\left(x\right)\cdot P_{Y|X}\left(y|x\right)$, where $P_{\hat{X}}\left(x\right)=\sum_{y}P_{\hat{X}\hat{Y}}\left(x,y\right)$ is the empirical marginal of $\hat{X}$. Such mixed joint distributions underlie certain information-theoretic quantities, for example, $I(\hat{X};Y)$ and $H(Y|\hat{X})$ denote, respectively, the mutual information between $\hat{X}$ and *Y* and the conditional entropy of *Y* given $\hat{X}$, both induced from $P_{\hat{X}Y}$. The same notation rules are applicable in more involved situations too.

### 2.2. Definitions and Problem Setting

**Figure 1:**
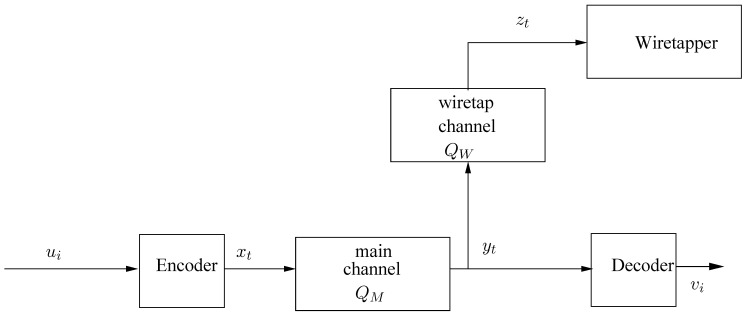
Wiretap channel model. Since the source and the channel may operate at different rates (λ channel symbols per source symbol), the time variables associated with source-related sequences and channel-related sequences are denoted differently, i.e., *i* and *t*, respectively.

We consider the system configuration of the degraded wiretap channel, depicted in Figure 1. Let $u=(u_{1},u_{2},\ldots )$ be a deterministic source sequence (i.e., individual sequence), whose symbols take values in a finite alphabet, U, of size α. This source sequence is to be conveyed reliably to a legitimate decoder while keeping it secret from a wiretapper, as described below. The encoding mechanism is as follows. The source sequence, ***u*** is first is divided into chunks of length *k*, ${\tilde{u}}_{i}=u_{ik+1}^{ik+k}\in U^{k}$, i=0,1,2,…, which are fed into a stochastic finite-state encoder, defined by the following equations:

(1) $$\begin{array}{cc} \mathrm{Pr}\{{\tilde{X}}_{i}=\tilde{x}|{\tilde{u}}_{i}=\tilde{u},\;s_{i}^{e}=s\} & =P(\tilde{x}|\tilde{u},s),\;\;\;\;i=0,1,2,\ldots \end{array}$$

(2) $$\begin{array}{cc} s_{i+1}^{e} & =h({\tilde{u}}_{i},s_{i}^{e}),\;\;\;\;i=0,1,2,\ldots . \end{array}$$

We allow a stochastic encoder, in view of the fact that even the traditional, probabilistic setting, optimal coding for the wiretap channel (see [1] and later works) must be randomized in order to meet the security requirements. Here, ${\tilde{X}}_{i}$ is a random vector taking values in $X^{m}$, X being the β-ary input alphabet of the channel and *m* being a positive integer; $\tilde{x}\in X^{m}$ is a realization of ${\tilde{X}}_{i}$; and $s_{i}^{e}$ is the state of the encoder at time *i*, which designates the memory of the encoder with regard to the past of the source sequence. In other words, at time instant *i*, whatever the encoder ‘remembers’ from $\left(u_{1},u_{2},\ldots ,u_{i-1}\right)$ is stored in the variable $s_{i}^{e}$ (for example, in the case of trellis coding, it can be a shift register of finite length, say *p*, that stores the most recent symbols of *p*, $\left(u_{i-p},u_{i-p+1},\ldots ,u_{i-1}\right)$, or, in block coding, it can be the contents of the current block starting from its beginning up to the present). The state, $s_{i}^{e}$, takes values in a finite set of states, $S^{e}$, of size $q_{e}$. In the above equation, the variable $\tilde{u}$ is any member of $U^{k}$. The function $h:U^{k}\times S^{e}\to S^{e}$ is called the *next-state function* of the encoder. (More generally, we could define both $s_{i+1}^{e}$ and ${\tilde{x}}_{i}$ to be random functions of the $\left({\tilde{u}}_{i},s_{i}^{e}\right)$ by a conditional joint distribution, $\mathrm{Pr}\{{\tilde{X}}_{i}=\tilde{x},s_{i+1}^{e}=s|{\tilde{u}}_{i}=\tilde{u},\;s_{i}^{e}=s^{\prime }\}$. However, it makes sense to let the encoder state sequence evolve deterministically in response to the input ***u*** since the state designates the memory of the encoder to past inputs.) Finally, $P(\tilde{x}|\tilde{u},s)$, $\tilde{u}\in U^{k}$, $s\in S^{e}$, $\tilde{x}\in X^{m}$, is a conditional probability distribution function, i.e., $\left\{P\left(\tilde{x}|\tilde{u},s\right)\right\}$ are all non-negative and $\sum_{\tilde{x}}P\left(\tilde{x}|\tilde{u},s\right)=1$ for all $\left(\tilde{u},s\right)\in U^{k}\times S^{e}$. The vector ${\tilde{x}}_{i}$ designates the current output vector from the encoder in response to the current input source vector, ${\tilde{u}}_{i}$ and its current state, $s_{i}^{e}$. Without loss of generality, we assume that the initial state of the encoder, $s_{0}^{e}$, is some fixed member of $S^{e}$. The ratio

(3) $$\lambda \overset{▵}{=}\frac{m}{k}$$

is referred to as the *bandwidth expansion factor*. It should be pointed out that the parameters *k* and *m* are fixed integers, which are not necessarily large (e.g., k=2 and m=3 are valid values of *k* and *m*). The concatenation of the output vectors from the encoder, ${\tilde{x}}_{0},{\tilde{x}}_{1},\ldots$, is viewed as a sequence chunks of channel input symbols, $x_{1},x_{2},\ldots$, with ${\tilde{x}}_{i}=x_{im+1}^{im+m}$, similarly to the above-defined partition of the source sequence.

The sequence of encoder outputs, $x_{1},x_{2},\ldots$, is fed into a discrete memoryless channel (DMC), henceforth referred to as the *main channel*, whose corresponding outputs, $y_{1},y_{2},\ldots$, are generated according to

(4) $$\mathrm{Pr}\left\{Y^{N}=y^{N}|X^{N}=x^{N}\right\}=Q_{M}\left(y^{N}|x^{N}\right)=\prod_{i=1}^{N}Q_{M}\left(y_{i}|x_{i}\right),$$

for every positive integer *N* and every $x^{N}\in X^{N}$ and $y^{N}\in Y^{N}$. The channel output symbols, $\left\{y_{i}\right\}$, take values in a finite alphabet, Y, of size γ.

The sequence of channel outputs, $y_{1},y_{2},\ldots$, is divided into chunks of length *m*, ${\tilde{y}}_{i}=y_{im+1}^{im+m}$, i=0,1,2,…, which are fed into a deterministic finite-state decoder, defined according to the following recursive equations:

(5) $$\begin{array}{ccc} {\tilde{v}}_{i} & = & f({\tilde{y}}_{i},s_{i}^{d}) \end{array}$$

(6) $$\begin{array}{ccc} s_{i+1}^{d} & = & g({\tilde{y}}_{i},s_{i}^{d}), \end{array}$$

where the variables in the equations are defined as follows: $\left\{s_{i}^{d}\right\}$ is the sequence of states of the decoder (which again, designate the finite memory, this time, at the decoder). Each $s_{i}^{d}$ takes values in a finite set, $S^{d}$ of size $q_{d}$. The variable ${\tilde{v}}_{i}\in U^{k}$ is the *i*-th chunk of *k* source reconstruction symbols, i.e., ${\tilde{v}}_{i}=v_{ik+1}^{ik+k}$, i=0,1,…, which form the decoder output. The function $f:Y^{m}\times S^{d}\to U^{k}$ is called the *output function* of the decoder and the function $g:Y^{m}\times S^{d}\to S^{d}$ is the next-state function of the decoder. The concatenation of the decoder output vectors, ${\tilde{v}}_{0},{\tilde{v}}_{1},\ldots$, forms the entire stream of reconstruction symbols, $v_{1},v_{2},\ldots$.

The output of the main channel, $y_{1},y_{2},\ldots$, is fed into another DMC, henceforth referred to as the *wiretap channel*, which generates, in response, a corresponding sequence, $z_{1},z_{2},\ldots$, according to

(7) $$\mathrm{Pr}\left\{Z^{N}=z^{N}|Y^{N}=y^{N}\right\}=Q_{W}\left(z^{N}|y^{N}\right)=\prod_{i=1}^{N}Q_{W}\left(z_{i}|y_{i}\right),$$

where $\left\{Z_{i}\right\}$ and $\left\{z_{i}\right\}$ take values in a finite alphabet Z. We denote the cascade of channels $Q_{M}$ and $Q_{W}$ by $Q_{MW}$, that is

(8) $$Q_{MW}\left(z|x\right)=\sum_{y\in Y}Q_{M}\left(y|x\right)Q_{W}\left(z|y\right).$$

We seek a communication system (P,h,f,g) which satisfies two requirements:

1. For a given $\epsilon_{r}>0$, the system satisfies the following reliability requirement: The bit error probability is guaranteed to be less than $\epsilon_{r}$, i.e.,
  (9) $$P_{b}\overset{▵}{=}\frac{1}{k}\sum_{i=1}^{k}\mathrm{Pr}\left\{V_{i}\neq u_{i}\right\}\leq \epsilon_{r}$$
  for every $\left(u_{1},\ldots ,u_{k}\right)$ and every combination of initial states of the encoder and the decoder, where Pr{·} is defined w.r.t. the randomness of the encoder and the main channel.
2. For a given $\epsilon_{s}>0$, the system satisfies the following security requirement: For every sufficiently large positive integer *n*,
  (10) $$\max_{\mu }I_{\mu }\left(U^{n};Z^{N}\right)\leq n\epsilon_{s},$$
  where N=nλ and $I_{\mu }\left(U^{n};Z^{N}\right)$ is the mutual information between $U^{n}$ and $Z^{N}$, induced by an input distribution $\mu =\{\mu \left(u^{n}\right),\;u^{n}\in U^{n}\}$ and the system, $\left\{P\left(z^{N}|u^{n}\right),\;u^{n}\in U^{n},z^{N}\in Z^{N}\right\}$.

As for the reliability requirement, note that the larger *k* is, the less stringent the requirement becomes. Concerning the security requirement, ideally, we would like to have perfect secrecy, which means that $P(z^{N}|u^{n})$ would be independent of $u^{n}$ (see also [20]), but it is more realistic to allow a small deviation from this idealization. This security metric is actually the maximum mutual information metric, or equivalently (see [15]) the semantic security, as mentioned in the Introduction.

### 2.3. Preliminaries and Background

We need two more definitions along with some background associated with them. The first is the *secrecy capacity* [1,14], which is the supremum of all coding rates for which there exist block codes that maintain both an arbitrarily small error probability at the legitimate decoder and an equivocation arbitrarily close to the unconditional entropy of the source. The secrecy capacity is given by

(11) $$C_{s}=\max_{P_{X}}I\left(X;Y|Z\right)=\max_{P_{X}}\left[I\left(X;Y\right)-I\left(X;Z\right)\right],$$

with $P_{XYZ}\left(x,y,z\right)=P_{X}\left(x\right)\times Q_{M}\left(y|x\right)Q_{W}\left(z|y\right)$ for all (x,y,z)∈X×Y×Z.

The second quantity we need to define is the LZ complexity [22]. In their famous paper [22], Ziv and Lempel actually developed a deterministic counterpart of source coding theory, where instead of imposing assumptions on probabilistic mechanisms that generate the data (i.e., memoryless sources, Markov sources, and general stationary sources), whose relevance to real-world data compression may be subject to dispute, they considered arbitrary, deterministic source sequences (i.e., individual sequences, in their terminology), but they imposed instead a limitation on the resources of the encoder (or the data compression algorithm): they assumed that it has limited storage capability (i.e., limited memory) of past data when encoding the current source symbol. This limited storage was modeled in terms of a finite-state machine, where the state variable of the encoder evolves recursively in time in response to the input and designates the information that the encoder ‘remembers’ from the past input (just like in the model description in Section 2.2 above). As mentioned earlier, a simple example of such a state variable can be the contents of a finite shift register, fed sequentially by the source sequence in which case the state contains a finite number of the most recent source symbols. This individual-sequence approach is appealing, because it is much more realistic to assume practical limitations on the encoder (which is under the control of the system designer) than to make assumptions on the statistics of the data to be compressed.

Ziv and Lempel developed an asymptotically optimal, practical compression algorithm (which is used in almost every computer), that is well known as the Lempel–Ziv (LZ) algorithm. This algorithm has several variants. One of them, which is called the LZ78 algorithm (where ‘78’ designates the year 1978), is based on the notion of *incremental parsing*: given the the source vector, $u^{n}$, the incremental parsing procedure sequentially parses this sequence into distinct phrases such that each new parsed phrase is the shortest string that has not been obtained before as a phrase, with a possible exception of the last phrase, which might be incomplete. Let $c(u^{n})$ denote the number of resulting phrases. For example, if n=10 and $u^{10}=\left(0000110110\right)$, then incremental parsing (from left to right) yields (0,00,01,1,011,0) and so, $c(u^{10})=6$. We define the *LZ complexity* of the individual sequence, $u^{n}$, as

(12) $$\rho_{\mathrm{LZ}}\left(u^{n}\right)\overset{▵}{=}\frac{c\left(u^{n}\right)\log c\left(u^{n}\right)}{n}.$$

As was shown by Ziv and Lempel in their seminal paper [22], for large *n*, the LZ complexity, $\rho_{\mathrm{LZ}}\left(u^{n}\right)$, is essentially the best compression ratio that can be achieved by any information lossless, finite-state encoder (up to some negligibly small terms, for large *n*), and it can be viewed as the individual-sequence analogue of the entropy rate.

## 3. Results

Before moving on to present our first main result, a simple comment is in order. Even in the traditional probabilistic setting, given a source with entropy *H* and a channel with capacity *C*, reliable communication cannot be accomplished unless H≤λC, where λ is the bandwidth expansion factor. Since both *H* and *C* are given and only λ is under the control of the system designer, it is natural to state this condition as a lower bound to bandwidth expansion factor, i.e., λ≥H/C. By the same token, in the presence of a secrecy constraint, λ must not fall below $H/C_{s}$. Our converse theorems for individual sequences are presented in the same spirit, where the entropy *H* at the numerator is replaced by an expression whose main term is the Lempel–Ziv compressibility.

We assume, without essential loss of generality, that *k* divides *n* (otherwise, omit the last (nmodk) symbols of $u^{n}$ and replace *n* by k·⌊n/k⌋ without affecting the asymptotic behavior as n→∞). Our first main result is the following.

**Theorem** **1.** *Consider the problem setting defined in Section 2. If there exists a stochastic encoder with $q_{e}$ states and a decoder with $q_{d}$ states that together satisfy the reliability constraint (9) and the security constraint (10), then the bandwidth expansion factor λ must be lower bounded as follows.*

(13) $$\lambda \geq \frac{\rho_{LZ}\left(u^{n}\right)-\Delta \left(\epsilon_{r}\right)-\epsilon_{s}-\zeta_{n}\left(q_{d},k\right)}{C_{s}},$$

*where*

(14) $$\Delta \left(\epsilon_{r}\right)\overset{▵}{=}h_{2}\left(\epsilon_{r}\right)+\epsilon_{r}\cdot \log\left(\alpha -1\right),$$

*with $h_{2}\left(\epsilon_{r}\right)=-\epsilon_{r}\log\epsilon_{r}-\left(1-\epsilon_{r}\right)\log\left(1-\epsilon_{r}\right)$ being the binary entropy function, and*

(15) $$\zeta_{n}\left(q_{d},k\right)=\min_{\left\{\ell \;divides\;n/k\right\}}\left[\frac{\log q_{d}+1}{k\ell }+\frac{2k\ell {\left(\log\alpha +1\right)}^{2}}{(1-\epsilon_{n})\log n}+\frac{2k\ell \alpha^{2k\ell }\log\alpha }{n}\right],$$

*with $\epsilon_{n}\to 0$ as n→∞.*

The proof of Theorem 1, like all other proofs in this article, is deferred to Section 5.

**Discussion.**  *A few comments are in order with regard to Theorem 1.*

1. *Irrelevance of $q_{e}$.* It is interesting to note that as far as the encoding and decoding resources are concerned, the lower bound depends on *k* and $q_{d}$, but not on the number of states of the encoder, $q_{e}$. This means that the same lower bound continues to hold, even if the encoder has an unlimited number of states. Pushing this to the extreme, even if the encoder has room to store the entire past, the lower bound of Theorem 1 would remain unaltered. The crucial bottleneck is, therefore, in the finite memory resources associated with the decoder, where the memory may help to reconstruct the source by exploiting empirical dependencies with the past. The dependence on $q_{e}$, however, appear later when we discuss local randomness resources as well as in the extension to the case of decoder side information.
2. *The redundancy term $\zeta_{n}\left(q_{d},k\right)$.* A technical comment is in order concerning the term $\zeta_{n}\left(q_{d},k\right)$, which involves minimization over all divisors of n/k, where we have already assumed that n/k is integer. Strictly speaking, if n/k happens to be a prime, this minimization is not very meaningful, as $\zeta_{n}\left(q_{d},k\right)$ would be relatively large. If this the case, a better bound is obtained if one omits some of the last symbols of $u^{n}$, thereby reducing *n* to, say, $n^{\prime }$ so that $n^{\prime }/k$ has a richer set of factors. Consider, for example, the choice $\ell =\ell_{n}=\left\lfloor \sqrt{\log n}\right\rfloor$ (instead of minimizing over *ℓ*) and replace n/k by the $n/k-(n/k\;\;\mathrm{mod}\;\;\ell_{n})$, without essential loss of tightness. This way, $\zeta_{n}\left(q_{d},k\right)$ would tend to zero as n→∞, for fixed *k* and $q_{d}$.
3. *Achievability.* Having established that $\zeta_{n}\left(q_{d},k\right)\to 0$, and given that $\epsilon_{r}$ and $\epsilon_{s}$ are small, it is clear that the main term at the numerator of the lower bound of Theorem 1 is the term $\rho_{\mathrm{LZ}}\left(u^{n}\right)$, which is, as mentioned earlier, the individual-sequence analogue of the entropy of the source [22]. In other words, λ cannot be much smaller than $\lambda_{L}\left(u^{n}\right)=\rho_{\mathrm{LZ}}\left(u^{n}\right)/C_{s}$. A matching achievability scheme would most naturally be based on separation: first apply variable-rate compression of $u^{n}$ to about $n\rho_{\mathrm{LZ}}\left(u^{n}\right)$ bits using the LZ algorithm [22], and then feed the resulting compressed bit-stream into a good code for the wiretap channel [1] with codewords of length about
  (16) $$N=n\lambda_{L}\left(u^{n}\right)∼\frac{n\rho_{\mathrm{LZ}}\left(u^{n}\right)}{C_{s}\left(1-\delta \right)},$$
  where δ is an arbitrarily small (but positive) margin to keep the coding rate strictly smaller than $C_{s}$. However, to this end, the decoder must know *N*. One possible solution is that before the actual encoding of each $u^{n}$, one would use a separate, auxiliary fixed code that encodes the value of the number of compressed bits, $n\rho_{\mathrm{LZ}}\left(u^{n}\right)$, using log(nlogα) bits (as nlogα is about the number of possible values that $n\rho_{\mathrm{LZ}}\left(u^{n}\right)$ can take) and protect it using a channel code of rate less than $C_{s}\left(1-\delta \right)$. Since the length of this auxiliary code grows only logarithmically with *n* (as opposed to the ‘linear’ growth of $n\rho_{\mathrm{LZ}}\left(u^{n}\right)$), the overhead in using the auxiliary code is asymptotically negligible. The auxiliary code and the main code are used alternately: first the auxiliary code, and then the main code for each *n*-tuple of the source. The main channel code is actually an array of codes, one for each possible value of $n\rho_{\mathrm{LZ}}\left(u^{n}\right)$. Once the auxiliary decoder has decoded this number, the corresponding main decoder is used. Overall, the resulting bandwidth expansion factor is about
  (17) $$\lambda \approx \frac{n\rho_{\mathrm{LZ}}\left(u^{n}\right)+\log\left(n\log\alpha \right)}{nC_{s}\left(1-\delta \right)}=\frac{\rho_{\mathrm{LZ}}\left(u^{n}\right)}{C_{s}\left(1-\delta \right)}+O\left(\frac{\log n}{n}\right).$$
  Another, perhaps simpler and better, approach is to use the LZ algorithm in the mode of a variable-to-fixed length code: let the length of the channel codeword, *N*, be fixed, and start to compress $u=(u_{1},u_{2},\ldots )$ until obtaining $n\rho_{\mathrm{LZ}}\left(u^{n}\right)=N\cdot C_{s}\left(1-\delta \right)$ compressed bits. Then,
  (18) $$\lambda =\frac{N}{n}=\frac{\rho_{\mathrm{LZ}}\left(u^{n}\right)}{C_{s}\left(1-\delta \right)}.$$
  Of course, these coding schemes require decoder memory that grows exponentially in *n*, and not just a fixed number, $q_{d}$, and therefore strictly speaking, there is a gap between the achievability and the converse result of Theorem 2. However, this gap is closed asymptotically, once we take the limit of $q_{d}\to 0$ after the limit n→∞, and we consider successive application of these codes over many blocks. The same approach appears also in [17,18,19,22] as well as in later related work.

This concludes the discussion on Theorem 1. □

We next focus on local randomness resources that are necessary when the full secrecy capacity is exploited. Specifically, suppose that the stochastic encoder $\left\{P\left(\tilde{x}|\tilde{u},s\right),\;\tilde{x}\in X^{n},\;\tilde{u}\in U^{k},\;s\in S^{e}\right\}$ is implemented as a deterministic encoder with an additional input of purely random bits, i.e.,

(19) $${\tilde{x}}_{i}=a\left({\tilde{u}}_{i},s_{i}^{e},{\tilde{b}}_{i}\right),$$

where ${\tilde{b}}_{i}=b_{ij+1}^{ij+j}$ is a string of *j* purely random bits. The question is the following: how large must *j* be in order to achieve full secrecy? Equivalently, what is the minimum necessary rate of random bits for local randomness at the encoder for secure coding at the maximum reliable rate? In fact, this question may be interesting on its own right, regardless of the individual-sequence setting and finite-state encoders and decoders, but even for ordinary block coding (which is the special case of $q_{e}=q_{d}=1$) and in the traditional probabilistic setting. The following theorem answers this question.

**Theorem** **2.** *Consider the problem setting defined in Section 2 and let λ meet the lower bound of Theorem 1. If there exists an encoder (19) with $q_{e}$ states and a decoder with $q_{d}$ states that jointly satisfy the reliability constraint (9) and the security constraint (10), then*

(20) $$j\geq mI\left(X^{*};Z^{*}\right)-k\epsilon_{s}-\frac{\log q_{e}}{\ell }$$

*where $X^{*}$ is the random variable that achieves $C_{s}$ and ℓ is the achiever of $\zeta_{n}\left(q_{d},k\right)$.*

Note that the lower bound of Theorem 2 depends on $q_{e}$, as opposed to Theorem 1, where it depends only on $q_{d}$. Since $\epsilon_{s}$ is assumed small and ℓ→∞, it is clear that main term is $mI(X^{*};Z^{*})$, i.e., the bit rate must be essentially at least as large as $I(X^{*};Z^{*})$ random bits per channel use, or equivalently, $\lambda I(X^{*};Z^{*})$ bits per source symbol. It is interesting to note that Wyner’s code [1] asymptotically achieves this bound when the coding rate saturates the secrecy capacity because the subcode that can be decoded by the wiretapper (within each given bin) is of the rate of about $I(X^{*};Z^{*})$, and it encodes just the bits of the local randomness. So when working at the full secrecy capacity, Wyner’s code is optimal not only in terms of the optimal trade-off between reliability and security, but also in terms of minimum consumption of local, purely random bits.

## 4. Side Information at the Decoder with Partial Leakage to the Wiretapper

**Figure 2:**
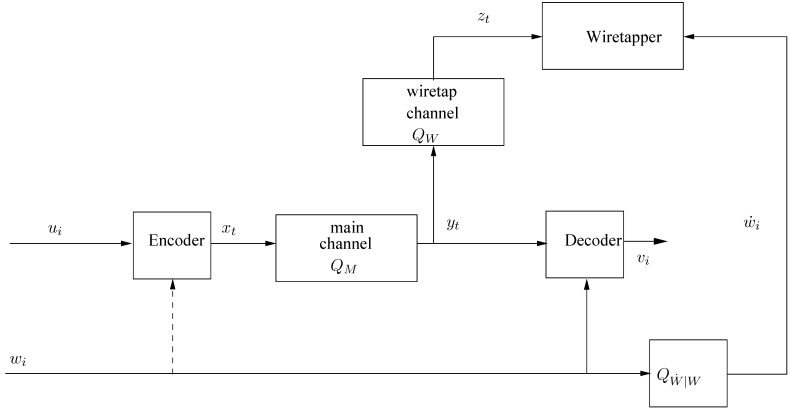
Wiretap channel model with side information.

Consider next an extension of our model to the case where there are side information sequences, $w^{n}=\left(w_{1},\ldots ,w_{n}\right)$ and ${\dot{w}}^{n}=\left({\dot{w}}_{1},\ldots ,{\dot{w}}_{n}\right)$, available to the decoder and the wiretapper, respectively; see Figure 2. For the purpose of a converse theorem, we assume that $w^{n}$ is available to the encoder too, whereas in the achievability part, we comment also on the case where it is not. We assume that $w^{n}$ is a deterministic sequence, but ${\dot{w}}^{n}$ is a realization of a random vector ${\dot{W}}^{n}=\left({\dot{W}}_{1},\ldots ,{\dot{W}}_{n}\right)$, which is a noisy version of $w^{n}$. In other words, it is generated from $w^{n}$ by another memoryless channel, $Q_{{\dot{W}}^{n}|W^{n}}\left({\dot{w}}^{n}|w^{n}\right)=\prod_{i=1}^{n}Q_{\dot{W}|W}\left({\dot{w}}_{i}|w_{i}\right)$. The symbols of $\left\{w_{i}\right\}$ and $\left\{{\dot{w}}_{i}\right\}$ take values in finite alphabets, W and $\dot{W}$, respectively. There are two extreme important special cases: (i) ${\dot{W}}^{n}=w^{n}$ almost surely, which is the case of totally insecure side information that fully leaks to the wiretapper, and (ii) ${\dot{W}}^{n}$ is degenerated (or independent of $w^{n}$), which is the case of secure side information with no leakage to the wiretapper. Every intermediate situation between these two extremes is a situation of partial leakage. The finite-state encoder model is now re-defined according to

(21) $$\begin{array}{ccc} \mathrm{Pr}\{{\tilde{X}}_{i}=\tilde{x}|{\tilde{u}}_{i}=\tilde{u},\;{\tilde{w}}_{i}=\tilde{w},s_{i}^{e}=s\} & = & P(\tilde{x}|\tilde{u},\tilde{w},s),\;\;\;\;i=0,1,2,\ldots \end{array}$$

(22) $$\begin{array}{ccc} s_{i+1}^{e} & = & h({\tilde{u}}_{i},{\tilde{w}}_{i},s_{i}^{e}),\;\;\;\;i=0,1,2,\ldots , \end{array}$$

where ${\tilde{w}}_{i}=w_{ik+1}^{ik+k}$, i=0,1,…,n/k−1. Likewise, the decoder is given by

(23) $$\begin{array}{ccc} {\tilde{v}}_{i} & = & f({\tilde{y}}_{i},{\tilde{w}}_{i},s_{i}^{d}) \end{array}$$

(24) $$\begin{array}{ccc} s_{i+1}^{d} & = & g({\tilde{y}}_{i},{\tilde{w}}_{i},s_{i}^{d}), \end{array}$$

and the wiretapper has access to $Z^{N}$ and ${\dot{W}}^{n}$. Accordingly, the security constraint is modified as follows: for a given $\epsilon_{s}>0$ and for every sufficiently large *n*,

(25) $$\max_{\mu }I_{\mu }\left(U^{n};Z^{N}|{\dot{W}}^{n}\right)\leq n\epsilon_{s},$$

where $I_{\mu }\left(U^{n};Z^{N}|{\dot{W}}^{n}\right)$ is the conditional mutual information between $U^{n}$ and $Z^{N}$ given ${\dot{W}}^{n}$, induced by $\mu =\{\mu \left(u^{n},{\dot{w}}^{n}\right),\;u^{n}\in U^{n},\;{\dot{w}}^{n}\in {\dot{W}}^{n}\}$ and the system, $\left\{P\left(z^{N}|u^{n}\right),\;u^{n}\in U^{n},z^{N}\in Z^{N}\right\}$, where $\mu \left(u^{n},{\dot{w}}^{n}\right)=\sum_{w^{n}}\mu \left(u^{n},w^{n}\right)Q_{{\dot{W}}^{n}|W^{n}}\left({\dot{w}}^{n}|w^{n}\right)$.

In order to present the extension of Theorem 1 to incorporate side information, we first need to define the extension of the LZ complexity to include side information, namely, to define the conditional LZ complexity (see also [23]). Given $u^{n}$ and $w^{n}$, let us apply the incremental parsing procedure of the LZ algorithm to the sequence of pairs $\left(\left(u_{1},w_{1}\right),\left(u_{2},w_{2}\right),\ldots ,\left(u_{n},w_{n}\right)\right)$. According to this procedure, all phrases are distinct with a possible exception of the last phrase, which might be incomplete. Let $c(u^{n},w^{n})$ denote the number of distinct phrases. As an example (which appears also in [23]), if

$$\begin{array}{ccc} u^{6} & = & 0\;|\;1\;|\;0\;0\;|\;0\;1|\\ w^{6} & = & 0\;|\;1\;|\;0\;1\;|\;0\;1| \end{array}$$

then $c(u^{6},w^{6})=4$. Let $c(w^{n})$ denote the resulting number of distinct phrases of $w^{n}$, and let w(l) denote the *l*-th distinct *w*-phrase, $l=1,2,...,c(w^{n})$. In the above example, $c(w^{6})=3$. Denote by $c_{l}\left(u^{n}|w^{n}\right)$ the number of occurrences of w(l) in the parsing of $w^{n}$, or equivalently, the number of distinct *u*-phrases that jointly appear with w(l). Clearly, $\sum_{l=1}^{c(w^{n})}c_{l}\left(u^{n}|w^{n}\right)=c\left(u^{n},w^{n}\right)$. In the above example, w(1)=0, w(2)=1, w(3)=01, $c_{1}\left(u^{6}|w^{6}\right)=c_{2}\left(u^{6}|w^{6}\right)=1$, and $c_{3}\left(u^{6}|w^{6}\right)=2$. Now, the conditional LZ complexity of $u^{n}$ given $w^{n}$ is defined as

(26) $$\rho_{LZ}\left(u^{n}|w^{n}\right)\overset{▵}{=}\frac{1}{n}\sum_{l=1}^{c(w^{n})}c_{l}\left(u^{n}|w^{n}\right)\log c_{l}\left(u^{n}|w^{n}\right).$$

We are now ready to present the main result of this section.

**Theorem** **3.** *Consider the problem setting defined in Section 2 along with the above–mentioned modifications to incorporate side information. If there exists a stochastic encoder with $q_{e}$ states and a decoder with $q_{d}$ states that together satisfy the reliability constraint (9) and the security constraint (25), then its bandwidth expansion factor λ must be lower bounded as follows.*

(27) $$\lambda \geq \frac{\rho_{LZ}\left(u^{n}|w^{n}\right)-\Delta \left(\epsilon_{r}\right)-\epsilon_{s}-\eta_{n}\left(q_{e}\cdot q_{d},k\right)}{C_{s}},$$

*where*

(28) $$\eta_{n}\left(q_{e}\cdot q_{d},k\right)=\min_{\left\{\ell \;divides\;n/k\right\}}\left[\frac{\log(q_{d}q_{e})+1}{k\ell }+\frac{\log(4A^{2})}{(1-\epsilon_{n})\log n}+\frac{A^{2}\log\left(4A^{2}\right)}{n}\right],$$

*with $\epsilon_{n}\to 0$ as n→∞ and $A=[{\left(\alpha \omega \right)}^{k\ell +1}-1]/\left[\alpha \omega -1\right]$, ω being the size of W.*

Note that the lower bound of Theorem 3 does not depend on the noisy side information at the wiretapper or on the channel $Q_{\dot{W}|W}$ that generates it from $w^{n}$. It depends only on $u^{n}$ and $w^{n}$ in terms of the data available in the system. Clearly, as it is a converse theorem, if it allows the side information to be available also at the encoder, then it definitely applies also to the case where the encoder does not have access to $w^{n}$. Interestingly, the encoder and the legitimate decoder act as if the wiretapper has the *clean* side information, $w^{n}$. While it is quite obvious that protection against availability of $w^{n}$ at the wiretapper is sufficient for protection against availability of ${\dot{W}}^{n}$ (as ${\dot{W}}^{n}$ is a degraded version of $w^{n}$), it is not quite trivial that this should be also *necessary*, as the above converse theorem asserts. It is also interesting to note that here, the bound depends also on $q_{e}$, and not only $q_{d}$, as in Theorem 1. However, this dependence on $q_{e}$ disappears in the special case where ${\dot{W}}^{n}=w^{n}$ with probability one.

We next discuss the achievability of the lower bound of Theorem 3. If the encoder has access to $w^{n}$, then the first step would be to apply the conditional LZ algorithm (see ([23], proof of Lemma 2) [24]), thus compressing $u^{n}$ to about $n\rho_{\mathrm{LZ}}\left(u^{n}|w^{n}\right)$ bits. The second step would be good channel coding for the wiretap channel, using the same methods as described in the previous section. If, however, the encoder does not have access to $w^{n}$, the channel coding part is still as before, but the situation with the source coding part is somewhat more involved since neither the encoder nor the decoder can calculate the target bit rate, $\rho_{\mathrm{LZ}}\left(u^{n}|w^{n}\right)$, as neither party has access to both $u^{n}$ and $w^{n}$. However, this source coding rate can essentially be achieved, provided that there is a low-rate noiseless feedback channel from the legitimate decoder to the encoder. The following scheme is in the spirit of the one proposed by Draper [25], but with a few modifications.

The encoder implements random binning for all source sequences in $U^{n}$, that is, for each member of $U^{n}$ an index is drawn independently, under the uniform distribution over $\left\{0,1,2,\ldots ,\alpha^{n}-1\right\}$, which is represented by its binary expansion, $b(u^{n})$, of length nlogα bits. We select a large positive integer *r*, but keep r≪n (say, $r=\sqrt{n}$ or $r=\log^{2}n$). The encoder transmits the bits of $b(u^{n})$ incrementally, *r* bits at a time, until it receives from the decoder ACK. Each chunk of *r* bits is fed into a good channel code for the wiretap channel, at a rate slightly less than $C_{s}$. At the decoder side, this channel code is decoded (correctly, with high probability, for large *r*). Then, for each *i* (i=1,2,…), after having decoded the *i*-th chunk of *r* bits of $b(u^{n})$, the decoder creates the list $A_{i}\left(u^{n}\right)=\left\{{\dot{u}}^{n}:\;{\left[b\left({\dot{u}}^{n}\right)\right]}^{ir}={\left[b\left(u^{n}\right)\right]}^{ir}\right\}$, where ${\left[b\left({\dot{u}}^{n}\right)\right]}^{l}$ denotes the string formed by the first *l* bits of $b({\dot{u}}^{n})$. For each ${\dot{u}}^{n}\in A_{i}\left(u^{n}\right)$, the decoder calculates $\rho_{\mathrm{LZ}}\left({\dot{u}}^{n}|w^{n}\right)$. We fix an arbitrarily small δ>0, which controls the trade-off between error probability and compression rate. If $n\rho_{\mathrm{LZ}}\left({\dot{u}}^{n}|w^{n}\right)\leq i\cdot r-n\delta$ for some ${\dot{u}}^{n}\in A_{i}\left(u^{n}\right)$, the decoder sends ACK on the feedback channel and outputs the reconstruction, ${\dot{u}}^{n}$, with the smallest $\rho_{\mathrm{LZ}}\left({\dot{u}}^{n}|w^{n}\right)$ among all members of $A_{i}\left(u^{n}\right)$. If no member of $A_{i}\left(u^{n}\right)$ satisfies $n\rho_{\mathrm{LZ}}\left({\dot{u}}^{n}|w^{n}\right)\leq i\cdot r-n\delta$, the receiver waits for the next chunk of *r* compressed bits, and it does not send ACK. The probability of source-coding error after the *i*-th chunk is upper bounded by

(29) $$\begin{array}{ccc} P_{e}\left(i\right) & \overset{\left(a\right)}{\leq } & |\left\{{\dot{u}}^{n}\neq u^{n}:\;n\rho_{\mathrm{LZ}}\left({\dot{u}}^{n}|w^{n}\right)\leq i\cdot r-n\delta \right\}|\cdot 2^{-i\cdot r}\\ & \overset{\left(b\right)}{\leq } & \exp_{2}\left\{i\cdot r-n\delta +O\left(\frac{n\log(\log n)}{\log n}\right)\right\}\cdot 2^{-i\cdot r}\\ & = & \exp_{2}\left\{-n\delta +O\left(\frac{n\log(\log n)}{\log n}\right)\right\}\\ & \to & 0\;\;\;\;as\;n\to \infty , \end{array}$$

where in (a), the factor $2^{-i\cdot r}$ is the probability that ${\left[b\left({\dot{u}}^{n}\right)\right]}^{ir}={\left[b\left(u^{n}\right)\right]}^{ir}$ for each member of the set $\left\{{\dot{u}}^{n}\neq u^{n}:\;n\rho_{\mathrm{LZ}}\left({\dot{u}}^{n}|w^{n}\right)\leq i\cdot r-n\delta \right\}$ and (b) is based on ([23], Equation (A.13)). Clearly, it is guaranteed that an ACK is received at the encoder (and hence the transmission stops), no later than after the transmission of chunk no. $i^{*}$, where $i^{*}$ is the smallest integer *i* such that $i\cdot r\geq n\rho_{\mathrm{LZ}}\left(u^{n}|w^{n}\right)+n\delta$, namely, $i^{*}=\left\lceil \left[n\rho_{\mathrm{LZ}}\left(u^{n}|w^{n}\right)+n\delta \right]/r\right\rceil$, which is the stage at which at least the correct source sequence begins to satisfy the condition $n\rho_{\mathrm{LZ}}\left(u^{n}|w^{n}\right)\leq i\cdot r-n\delta$. Therefore, the compression ratio is no worse than $i^{*}\cdot r/n=\left\lceil n\left[\rho_{\mathrm{LZ}}\left(u^{n}|w^{n}\right)+\delta \right]/r\right\rceil \cdot r/n\leq \rho_{\mathrm{LZ}}\left(u^{n}|w^{n}\right)+\delta +r/n$. The overall probability of source-coding error is then upper bounded by

(30) $$P_{e}=\mathrm{Pr}\overset{i^{*}}{\underset{i=1}{⋃}}\left\{\mathrm{error}\;\mathrm{at}\;\mathrm{state}\;i\right\}\leq \sum_{i=1}^{i^{*}}P_{e}\left(i\right)\leq \left(\frac{n\log\alpha }{r}+1\right)\cdot \exp_{2}\left\{-n\delta +O\left(\frac{n\log(\log n)}{\log n}\right)\right\},$$

which still tends to zero as n→∞. As for channel-coding errors, the probability that at least one chunk is decoded incorrectly is upper bounded by $\left(\frac{n\log\alpha }{r}+1\right)\cdot e^{-rE}$, where *E* is an achievable error exponent of channel coding at the given rate. Thus, if *r* grows at any rate faster than logarithmic, but sub-linearly in *n*, then the overall channel-coding error probability tends to zero and, at the same time, the compression redundancy, r/n, tends to zero too.

To show that the security constraint (25) is satisfied too, consider an arbitrary assignment μ of random vectors $\left(U^{n},W^{n}\right)$, and let us denote by *B* the string of $I(X^{N};Z^{N})-N\epsilon$ bits of local randomness in Wyner’s code [1]. Then,

(31) $$\begin{array}{ccc} I(X^{N};Z^{N}) & = & H\left(Z^{N}\right)-H\left(Z^{N}|X^{N}\right)\\ & \overset{\left(a\right)}{\geq } & H\left(Z^{N}\right)-H\left(Z^{N}|U^{n},B\right)\\ & \overset{\left(b\right)}{\geq } & H\left(Z^{N}|{\dot{W}}^{n}\right)-H\left(Z^{N}|U^{n},B\right)\\ & \overset{\left(c\right)}{=} & H\left(Z^{N}|{\dot{W}}^{n}\right)-H\left(Z^{N}|U^{n},B,{\dot{W}}^{n}\right)\\ & = & I(U^{n},B;Z^{N}|{\dot{W}}^{n})\\ & = & H\left(U^{n},B|{\dot{W}}^{n}\right)-H\left(U^{n},B|Z^{n},{\dot{W}}^{n}\right)\\ & = & H\left(U^{n}|{\dot{W}}^{n}\right)+H\left(B|U^{n},{\dot{W}}^{n}\right)-H\left(U^{n}|Z^{N},{\dot{W}}^{n}\right)-H\left(B|Z^{N},{\dot{W}}^{n},U^{n}\right)\\ & \overset{\left(d\right)}{=} & H\left(U^{n}|{\dot{W}}^{n}\right)+H\left(B\right)-H\left(U^{n}|Z^{N},{\dot{W}}^{n}\right)-H\left(B|Z^{N},{\dot{W}}^{n},U^{n}\right)\\ & \overset{\left(e\right)}{\geq } & H\left(U^{n}|{\dot{W}}^{n}\right)+H\left(B\right)-H\left(U^{n}|Z^{N},{\dot{W}}^{n}\right)-H\left(B|Z^{N},U^{n}\right)\\ & \overset{\left(f\right)}{\geq } & H\left(U^{n}|{\dot{W}}^{n}\right)+\left[I\left(X^{N};Z^{N}\right)-N\epsilon \right]-H\left(U^{n}|Z^{N},{\dot{W}}^{n}\right)-n\delta_{n}\\ & = & I\left(X^{N};Z^{N}\right)+I_{\mu }\left(U^{n};Z^{N}|{\dot{W}}^{n}\right)-n\left(\lambda \epsilon +\delta_{m}\right), \end{array}$$

where (a) is due to $\left(U^{n},B\right)\to X^{N}\to Z^{N}$ being a Markov chain, (b) is due to conditioning reducing entropy, (c) is due to ${\dot{W}}^{n}\to \left(U^{n},B\right)\to Z^{N}$ being a Markov chain, (d) is due to *B* being independent of $\left(U^{n},{\dot{W}}^{n}\right)$, (e) is due to conditioning reducing entropy, and (f) is due to, in Wyner coding, *B* being able to be reliably decoded given that $\left(Z^{N},U^{n}\right)$ ($\delta_{n}$ is understood to be small, and recall that $W^{n}$ is not needed in the channel decoding phase, but only in the Slepian–Wold decoding phase), and that the length of *B* is chosen to be $I(X^{N};Z^{N})-N\epsilon$. Comparing the right-most side to the left-most side, we readily obtain

(32) $$I_{\mu }\left(U^{n};Z^{N}|{\dot{W}}^{n}\right)\leq n\left(\lambda \epsilon +\delta_{n}\right),$$

which can be made arbitrarily small.

## 5. Proofs

We begin this section by establishing more notation conventions to be used throughout all proofs.

Let n≫k be a positive integer and let *ℓ* be such that $K\overset{▵}{=}\ell \cdot k$ divides *n*. Consider the partition of $u^{n}$ into n/K non-overlapping blocks of length *K*,

(33) $$\begin{array}{ccc} & & \left({\tilde{u}}_{0},{\tilde{u}}_{1},\ldots ,{\tilde{u}}_{\ell -1}\right),\left({\tilde{u}}_{\ell },{\tilde{u}}_{\ell +1},\ldots ,{\tilde{u}}_{2\ell -1}\right),\ldots ,\left({\tilde{u}}_{n/k-\ell },{\tilde{u}}_{n/k-\ell +1},{\tilde{u}}_{n/k-1}\right)\\ & = & \left(u_{1}^{K},u_{K+1}^{2K},\ldots ,u_{n-K+1}^{n}\right) \end{array}$$

and apply the same partition to $v^{n}$. The corresponding channel input and output sequences are of length N=nλ. Let M=ℓ·m=Kλ and consider the parallel partition of the channels input and output sequences according to

(34) $$\begin{array}{ccc} & & \left({\tilde{x}}_{0},{\tilde{x}}_{1},\ldots ,{\tilde{x}}_{\ell -1}\right),\left({\tilde{x}}_{\ell },{\tilde{x}}_{\ell +1},\ldots ,{\tilde{x}}_{2\ell -1}\right),\ldots ,\left({\tilde{x}}_{N/m-\ell },{\tilde{x}}_{N/m-\ell +1},\ldots ,{\tilde{x}}_{N/m-1}\right)\\ & & \left({\tilde{y}}_{0},{\tilde{y}}_{1},\ldots ,{\tilde{y}}_{\ell -1}\right),\left({\tilde{y}}_{\ell },{\tilde{y}}_{\ell +1},\ldots ,{\tilde{y}}_{2\ell -1}\right),\ldots ,\left({\tilde{y}}_{N/m-\ell },{\tilde{y}}_{N/m-\ell +1},\ldots ,{\tilde{y}}_{N/m-1}\right)\\ & & \left({\tilde{z}}_{0},{\tilde{z}}_{1},\ldots ,{\tilde{z}}_{\ell -1}\right),\left({\tilde{z}}_{\ell },{\tilde{z}}_{\ell +1},\ldots ,{\tilde{z}}_{2\ell -1}\right),\ldots ,\left({\tilde{z}}_{N/m-\ell },{\tilde{z}}_{N/m-\ell +1},\ldots ,{\tilde{z}}_{N/m-1}\right). \end{array}$$

For the sake of brevity, we henceforth denote $\left({\tilde{u}}_{i\ell },\ldots ,{\tilde{u}}_{(i+1)\ell -1}\right)$ by ${\tilde{u}}_{i\ell }^{(i+1)\ell -1}$ and use the same notation rule for all other sequences. Next, define the joint empirical distribution

(35) $$\begin{array}{ccc} & & P_{{\hat{U}}^{K}{\hat{X}}^{M}{\hat{Y}}^{M}{\hat{Z}}^{M}{\hat{S}}^{e}{\hat{S}}^{d}}\left(u^{K},x^{M},y^{M},z^{M},s^{e},s^{d}\right)=\\ & & \frac{K}{n}\sum_{i=0}^{n/K-1}\delta \{{\tilde{u}}_{i\ell }^{(i+1)\ell -1}=u^{K},{\tilde{x}}_{i\ell }^{(i+1)\ell -1}=x^{M},{\tilde{y}}_{i\ell }^{(i+1)\ell -1}=y^{M},\\ & & {\tilde{z}}_{i\ell }^{(i+1)\ell -1}=z^{M},s_{i\ell +1}^{e}=s^{e},s_{i\ell +1}^{d}=s^{d}\}, \end{array}$$

and

(36) $$P_{{\hat{U}}^{K}X^{M}Y^{M}Z^{M}{\hat{S}}^{e}S^{d}}\left(u^{K},x^{M},y^{M},z^{M},s^{e},s^{d}\right)=E\left\{P_{{\hat{U}}^{K}{\hat{X}}^{M}{\hat{Y}}^{M}{\hat{Z}}^{M}{\hat{S}}^{e}{\hat{S}}^{d}}\left(u^{K},x^{M},y^{M},z^{M},s^{e},s^{d}\right)\right\},$$

where the expectation is w.r.t. both the randomness of the encoder and the randomness of both channels. Note that

(37) $$P_{{\hat{U}}^{K}X^{M}Y^{M}Z^{M}{\hat{S}}^{e}}\left(u^{K},x^{M},y^{M},z^{M},s^{e}\right)=P_{{\hat{U}}^{K}{\hat{S}}^{e}}\left(u^{K},s^{e}\right)P\left(x^{M}|u^{K},s^{e}\right)Q_{M}\left(y^{M}|x^{M}\right)Q_{W}\left(z^{M}|y^{M}\right).$$

where

(38) $$\begin{array}{ccc} P(x^{M}|u^{K},s^{e}) & = & \prod_{j=0}^{\ell -1}P\left({\tilde{x}}_{j}|{\tilde{u}}_{j},s_{j}^{e}\right),\;\;\;\;s_{0}^{e}=s^{e} \end{array}$$

(39) $$\begin{array}{ccc} Q_{M}\left(y^{M}|x^{M}\right) & = & \prod_{j=0}^{M-1}Q_{M}\left(y_{i}|x_{i}\right) \end{array}$$

(40) $$\begin{array}{ccc} Q_{W}\left(z^{M}|y^{M}\right) & = & \prod_{j=0}^{M-1}Q_{M}\left(z_{i}|y_{i}\right). \end{array}$$

Note also that the bit error probability (in the absence of side information) under this distribution is

(41) $$\begin{array}{ccc} & & \frac{1}{K}E\left\{d_{H}\left({\hat{U}}^{K},f\left(Y^{M},S^{d}\right)\right)\right\}\\ & = & \frac{1}{K}\sum_{u^{K},y^{M},s^{e},s^{d}}P_{{\hat{U}}^{K}Y^{M}S^{e}S^{d}}\left(u^{K},y^{M},s^{e},s^{d}\right)d_{H}\left(u^{K},f\left(y^{M},s^{d}\right)\right)\\ & = & \frac{1}{K}\sum_{u^{K},y^{M},s^{d}}\frac{K}{n}\sum_{i=0}^{n/K-1}E\left[\delta \{{\tilde{u}}_{i\ell }^{(i+1)\ell -1}=u^{K},s_{i\ell +1}^{e}=s^{e},{\tilde{y}}_{i\ell }^{(i+1)\ell -1}=y^{M},s_{i\ell +1}^{d}=s^{d}\right]\times \\ & & d_{H}\left(u^{K},f\left(y^{M},s^{d}\right)\right)\\ & = & \frac{1}{n}\sum_{i=0}^{n/K-1}\sum_{y^{M},s^{d}}F\left(y^{M},s^{d}|u_{iK+1}^{iK+K},s_{i\ell +1}^{e}\right)d_{H}\left(u_{iK+1}^{iK+K},f\left(y^{M},s^{d}\right)\right)\\ & = & \frac{1}{n}\sum_{i=1}^{n}E\left\{d_{H}\left(u_{i},V_{i}\right)\right\}, \end{array}$$

where $f(Y^{M},S^{d})$ is induced by *ℓ* successive applications of the decoder output function with inputs $Y^{m},Y_{m+1}^{2m},\ldots ,Y_{M-m+1}^{M}$ and the initial state $S^{d}$, and where

(42) $$F\left(y^{M},s^{d}|u^{K},s^{e}\right)=\sum_{x^{M}}P\left(x^{M}|u^{K},s^{e}\right)Q_{M}\left(y^{M}|x^{M}\right)P_{S^{d}|Y^{M}}\left(s^{d}|y^{M}\right).$$

### 5.1. Proof of Theorem 1

Beginning with the reliability constraint, we have

(43) $$\begin{array}{ccc} I({\hat{U}}^{K};Y^{M},S^{d}) & = & H\left({\hat{U}}^{K}\right)-H\left({\hat{U}}^{K}|Y^{M},S^{d}\right)\\ & = & H\left({\hat{U}}^{K}\right)-H\left({\hat{U}}^{K}|Y^{M}\right)+I\left(S^{d};{\hat{U}}^{K}|Y^{M}\right)\\ & \leq & I\left({\hat{U}}^{K};Y^{M}\right)+H\left(S^{d}|Y^{M}\right)\\ & \leq & I\left(X^{M};Y^{M}\right)+\log q_{d}.\; \end{array}$$

On the other hand,

(44) $$\begin{array}{ccc} I({\hat{U}}^{K};Y^{M},S^{d}) & = & H\left({\hat{U}}^{K}\right)-H\left({\hat{U}}^{K}|Y^{M},S^{d}\right)\\ & \geq & H\left({\hat{U}}^{K}\right)-K\Delta \left(\epsilon_{r}\right), \end{array}$$

and so,

(45) $$\begin{array}{ccc} I(X^{M};Y^{M}) & \geq & H\left({\hat{U}}^{K}\right)-K\Delta \left(\epsilon_{r}\right)-\log q_{d}\\ & \overset{▵}{=} & K\cdot R(u^{n},q_{d},\epsilon_{r})\\ & = & M\cdot \frac{R(u^{n},q_{d},\epsilon_{r})}{\lambda }. \end{array}$$

Following [1], we define the function

(46) $$\Gamma \left[R\right]=\max_{\left\{P_{X}:\;I\left(X;Y\right)\geq R\right\}}I\left(X;Y|Z\right)=\max_{\left\{P_{X}:\;I\left(X;Y\right)\geq R\right\}}\left[I\left(X;Y\right)-I\left(X;Z\right)\right],$$

which is monotonically non–increasing and concave ([1], Lemma 1). Regarding the security constraint,

(47) $$\begin{array}{ccc} H\left({\hat{U}}^{K}\right)-K\epsilon_{s} & \overset{\left(a\right)}{\leq } & H\left({\hat{U}}^{K}\right)-\max_{\mu }I_{\mu }\left(U^{K};Z^{M}\right)\\ & \leq & H\left({\hat{U}}^{K}\right)-I\left({\hat{U}}^{K};Z^{M}\right)\\ & = & H\left({\hat{U}}^{K}|Z^{M}\right)-H\left({\hat{U}}^{K}|Y^{M},Z^{M},S^{d}\right)+H\left({\hat{U}}^{K}|Y^{M},Z^{M},S^{d}\right)\\ & = & H\left({\hat{U}}^{K}|Z^{M}\right)-H\left({\hat{U}}^{K}|Y^{M},Z^{M}\right)+I\left(S^{d};{\hat{U}}^{K}|Y^{M},Z^{M}\right)+H\left({\hat{U}}^{K}|Y^{M},Z^{M},S^{d}\right)\\ & \overset{\left(b\right)}{\leq } & I\left({\hat{U}}^{K};Y^{M}|Z^{M}\right)+\log q_{d}+K\Delta \left(\epsilon_{r}\right)\\ & \overset{\left(c\right)}{\leq } & I\left(X^{M};Y^{M}|Z^{M}\right)+\log q_{d}+K\Delta \left(\epsilon_{r}\right)\\ & \overset{\left(d\right)}{\leq } & \sum_{i=1}^{M}I\left(X_{i};Y_{i}|Z_{i},Y^{i-1}\right)+\log q_{d}+K\Delta \left(\epsilon_{r}\right)\\ & = & \sum_{i=1}^{M}\sum_{y^{i-1}}P_{Y^{i-1}}\left(y^{i-1}\right)I\left(X_{i};Y_{i}|Z_{i},Y^{i-1}=y^{i-1}\right)+\log q_{d}+K\Delta \left(\epsilon_{r}\right)\\ & \overset{\left(e\right)}{\leq } & M\cdot \frac{1}{M}\sum_{i=1}^{M}\sum_{y^{i-1}}P_{Y^{i-1}}\left(y^{i-1}\right)\Gamma \left[I\left(X_{i};Y_{i}|Y^{i-1}=y^{i-1}\right)\right]+\log q_{d}+K\Delta \left(\epsilon_{r}\right)\\ & \overset{\left(f\right)}{\leq } & M\cdot \Gamma \left[\frac{1}{M}\sum_{i=1}^{M}\sum_{y^{i-1}}P_{Y^{i-1}}\left(y^{i-1}\right)I\left(X_{i};Y_{i}|Y^{i-1}=y^{i-1}\right)\right]+\log q_{d}+K\Delta \left(\epsilon_{r}\right)\\ & = & M\cdot \Gamma \left[\frac{1}{M}\sum_{i=1}^{M}I\left(X_{i};Y_{i}|Y^{i-1}\right)\right]+\log q_{d}+K\Delta \left(\epsilon_{r}\right)\\ & = & M\cdot \Gamma \left[\frac{1}{M}\sum_{i=1}^{M}\left\{H\left(Y_{i}|Y^{i-1}\right)-H\left(Y_{i}|X_{i},Y^{i-1}\right)\right\}\right]+\log q_{d}+K\Delta \left(\epsilon_{r}\right)\\ & = & M\cdot \Gamma \left[\frac{1}{M}\sum_{i=1}^{M}\left\{H\left(Y_{i}|Y^{i-1}\right)-H\left(Y_{i}|X_{i}\right)\right\}\right]+\log q_{d}+K\Delta \left(\epsilon_{r}\right)\\ & = & M\cdot \Gamma \left[\frac{1}{M}\left\{H\left(Y^{M}\right)-H\left(Y^{M}|X^{M}\right)\right\}\right]+\log q_{d}+K\Delta \left(\epsilon_{r}\right)\\ & = & M\cdot \Gamma \left[\frac{I(X^{M};Y^{M})}{M}\right]+\log q_{d}+K\Delta \left(\epsilon_{r}\right)\\ & \overset{\left(g\right)}{\leq } & M\cdot \Gamma \left[\frac{R(u^{n},q_{d},\epsilon_{r})}{\lambda }\right]+\log q_{d}+K\Delta \left(\epsilon_{r}\right)\\ & \leq & M\cdot \Gamma \left[\frac{R\left(u^{n},q_{d},\epsilon_{r}\right)-\epsilon_{s}}{\lambda }\right]+\log q_{d}+K\Delta \left(\epsilon_{r}\right), \end{array}$$

where $P_{Y^{i-1}}\left(y^{i-1}\right)=\sum_{y_{i}^{M}}P_{Y^{M}}\left(y^{M}\right)$, (a) is due to the security constraint, (b) follows from Fano’s inequality and the fact that $I\left(S^{d};{\hat{U}}^{K}|Y^{M},Z^{M}\right)\leq H\left(S^{d}\right)\leq \log q_{d}$, (c) is due to the data processing inequality and the fact that ${\hat{U}}^{K}\to X^{M}\to Y^{M}$ is a Markov chain given $Z^{M}$, (d) is as in ([1], Equation (37)), (e) is by the definition of Wyner’s function Γ(·), (f) is by the concavity of this function, and (g) is by (45) and the decreasing monotonicity of the function Γ(·). Thus,

(48) $$\frac{R\left(u^{n},q_{d},\epsilon_{r}\right)-\epsilon_{s}}{\lambda }\leq \Gamma \left[\frac{R\left(u^{n},q_{d},\epsilon_{r}\right)-\epsilon_{s}}{\lambda }\right]$$

or

(49) $$\frac{R\left(u^{n},q_{d},\epsilon_{r}\right)-\epsilon_{s}}{\lambda }\leq C_{s}$$

which is

(50) $$R\left(u^{n},q_{d},\epsilon_{r}\right)\leq \lambda C_{s}+\epsilon_{s}$$

or, equivalently,

(51) $$\frac{H({\hat{U}}^{K})}{K}\leq \lambda C_{s}+\epsilon_{s}+\Delta \left(\epsilon_{r}\right)+\frac{\log q_{d}}{K}.$$

Finally, we apply the inequality ([20], Equation (18))

(52) $$\frac{H({\hat{U}}^{K})}{K}\geq \rho_{\mathrm{LZ}}\left(u^{n}\right)-\frac{2K{\left(\log\alpha +1\right)}^{2}}{(1-\epsilon_{n})\log n}-\frac{2K\alpha^{2K}\log\alpha }{n}-\frac{1}{K},$$

to obtain

(53) $$\rho_{\mathrm{LZ}}\left(u^{n}\right)\leq \lambda C_{s}+\epsilon_{s}+\Delta \left(\epsilon_{r}\right)+\zeta_{n}\left(q_{d},k\right),$$

which completes the proof of Theorem 1.

### 5.2. Proof of Theorem 2

Consider the following extension of the joint distribution to include a random variable that represents $\left\{b_{i}\right\}$, as follows:

(54) $$\begin{array}{ccc} & & P_{{\hat{U}}^{K}B^{J}X^{M}Y^{M}Z^{M}{\hat{S}}^{e}S^{d}}\left(u^{K},b^{J},x^{M},y^{M},z^{M},s^{e},s^{d}\right)=\\ & & \frac{K}{n}\sum_{i=0}^{n/K-1}E[\delta \{{\tilde{u}}_{i\ell }^{(i+1)\ell -1}=u^{K},{\tilde{b}}_{i\ell }^{(i+1)\ell -1}=b^{J},{\tilde{x}}_{i\ell }^{(i+1)\ell -1}=x^{M},{\tilde{y}}_{i\ell }^{(i+1)\ell -1}=y^{M},\\ & & {\tilde{z}}_{i\ell }^{(i+1)\ell -1}=z^{M},s_{i\ell +1}^{e}=s^{e},s_{i\ell +1}^{d}=s^{d}\}], \end{array}$$

where J=jℓ and ${\tilde{b}}_{i\ell }^{(i+1)\ell -1}=\left({\tilde{b}}_{i\ell },{\tilde{b}}_{i\ell +1},\ldots ,{\tilde{b}}_{(i+1)\ell -1}\right)$. Next, consider the following chain of inequalities

(55) $$\begin{array}{ccc} K\epsilon_{s} & \geq & \max_{\mu }I_{\mu }\left(U^{K};Z^{M}\right)\\ & \geq & I({\hat{U}}^{K};Z^{M})\\ & = & I\left({\hat{U}}^{K},B^{J},S^{e};Z^{M}\right)-I\left(B^{J},S^{e};Z^{M}|{\hat{U}}^{K}\right)\\ & \overset{\left(a\right)}{=} & I\left(X^{M};Z^{M}\right)-I\left(B^{J},S^{e};Z^{M}|{\hat{U}}^{K}\right)\\ & \geq & I\left(X^{M};Z^{M}\right)-H\left(B^{J},S^{e}|{\hat{U}}^{K}\right)\\ & \geq & I\left(X^{M};Z^{M}\right)-H\left(B^{J},S^{e}\right)\\ & \geq & I\left(X^{M};Z^{M}\right)-H\left(B^{J}\right)-H\left(S^{e}\right)\\ & \geq & I\left(X^{M};Z^{M}\right)-J-\log q_{e}, \end{array}$$

where (a) is due to the fact that, on the one hand, $X^{M}$ is a deterministic function of $\left({\hat{U}}^{K},B^{J},S^{e}\right)$, which implies that $I\left({\hat{U}}^{K},B^{J},S^{e};Z^{M}\right)\geq I\left(X^{M};Z^{M}\right)$, but on the other hand, $\left({\hat{U}}^{K},B^{J},S^{e}\right)\to X^{M}\to Z^{M}$ is a Markov chain and so, $I\left({\hat{U}}^{K},B^{J},S^{e};Z^{M}\right)\leq I\left(X^{M};Z^{M}\right)$, hence the equality. Thus,

(56) $$J\geq I\left(X^{M};Z^{M}\right)-K\epsilon_{s}-\log q_{e},$$

or

(57) $$j\geq \frac{I(X^{M};Z^{M})}{\ell }-k\epsilon_{s}-\frac{\log q_{e}}{\ell }\geq \frac{mI(X^{M};Z^{M})}{M}-k\epsilon_{s}-\frac{\log q_{e}}{\ell }.$$

The meaning of this result is the following: once one finds a communication system that complies with both the security constraint and the reliability constraint, then the amount of local randomization is lower bounded in terms of the induced mutual information, $I(X^{M};Z^{M})$, as above. By the hypothesis of Theorem 2, the secrecy capacity is saturated, and hence $P_{X^{M}}$ must coincide with the product distribution, ${\left[P_{X^{*}}\right]}^{M}$, yielding $I\left(X^{M};Z^{M}\right)/M=I\left(X^{*};Z^{*}\right)$. Thus,

(58) $$j\geq mI\left(X^{*};Z^{*}\right)-k\epsilon_{s}-\frac{\log q_{e}}{\ell }.$$

This completes the proof of Theorem 2.

### 5.3. Outline of the Proof of Theorem 3

The proof follows essentially the same steps as those of the proof of Theorem 1, except that everything should be conditioned on the side information, but there are also some small twists. We, therefore, only provide a proof outline and highlight the differences.

The auxiliary joint distribution is now extended to read

(59) $$\begin{array}{ccc} & & P_{{\hat{U}}^{K}{\hat{W}}^{K}{\dot{W}}^{K}X^{N}Y^{N}Z^{N}{\hat{S}}^{e}S^{d}}\left(u^{K},w^{K},{\dot{w}}^{K},x^{N},y^{N},z^{N},s^{e},s^{d}\right)=\\ & & \frac{K}{m}\sum_{i=0}^{m/K-1}E[\delta \{{\tilde{u}}_{i\ell }^{(i+1)\ell -1}=u^{K},{\tilde{w}}_{i\ell }^{(i+1)\ell -1}=w^{K},{\tilde{\dot{w}}}_{i\ell }^{(i+1)\ell -1}={\dot{w}}^{K},{\tilde{x}}_{i\ell }^{(i+1)\ell -1}=x^{M},\\ & & {\tilde{y}}_{i\ell }^{(i+1)\ell -1}=y^{M},{\tilde{z}}_{i\ell }^{(i+1)\ell -1}=z^{M},s_{i\ell +1}^{e}=s^{e},s_{i\ell +1}^{d}=s^{d}\}]. \end{array}$$

Note that

(60) $$\begin{array}{ccc} & & P_{{\hat{U}}^{K}{\hat{W}}^{K}{\dot{W}}^{K}Z^{M}{\hat{S}}^{e}}\left(u^{k},w^{k},{\dot{w}}^{k},z^{M},s^{e}\right)\\ & = & \frac{K}{n}\sum_{i=0}^{n/K-1}E\left[\delta \{{\tilde{u}}_{i\ell }^{(i+1)\ell -1}=u^{K},{\tilde{w}}_{i\ell }^{(i+1)\ell -1}=w^{K},{\tilde{\dot{w}}}_{i\ell }^{(i+1)\ell -1}={\dot{w}}^{K},{\tilde{z}}_{i\ell }^{(i+1)\ell -1}=z^{M},s_{i\ell +1}^{e}=s^{e}\}\right]\\ & = & \frac{K}{n}\sum_{i=0}^{n/K-1}\delta \left\{{\tilde{u}}_{i\ell }^{(i+1)\ell -1}=u^{K},{\tilde{w}}_{i\ell }^{(i+1)\ell -1}=w^{K},s_{i\ell +1}^{e}=s^{e}\right\}\cdot Q_{{\dot{W}}^{K}|W^{K}}\left({\dot{w}}^{K}|w^{K}\right)\cdot G\left(z^{M}|u^{K},s^{e}\right)\\ & = & P_{{\hat{U}}^{K}{\hat{W}}^{K}{\hat{S}}^{e}}\left(u^{K},w^{K},s^{e}\right)\cdot Q_{{\dot{W}}^{K}|W^{K}}\left({\dot{w}}^{K}|w^{K}\right)\cdot G\left(z^{M}|u^{K},s^{e}\right), \end{array}$$

where

(61) $$G\left(z^{M}|u^{K},s^{e}\right)=\sum_{x^{M}}P\left(x^{M}|u^{K},s^{e}\right)Q_{MW}\left(z^{M}|x^{M}\right).$$

It follows that ${\dot{W}}^{K}\to {\hat{W}}^{K}\to \left({\hat{U}}^{K},{\hat{S}}^{e}\right)\to Z^{M}$ is a Markov chain under $P_{{\hat{U}}^{K}{\hat{W}}^{K}{\dot{W}}^{K}Z^{M}{\hat{S}}^{e}}$. In other words, the legitimate decoder has side information of better quality than that of the wiretapper. First, observe that

(62) $$\begin{array}{ccc} I_{\mu }\left(U^{n};Z^{N}|W^{n}\right) & = & H_{\mu }\left(Z^{N}|W^{n}\right)-H_{\mu }\left(Z^{N}|W^{n},U^{n}\right)\\ & \leq & H_{\mu }\left(Z^{N}|W^{n}\right)-H_{\mu }\left(Z^{N}|W^{n},U^{n},S^{e}\right)\\ & = & H_{\mu }\left(Z^{N}|W^{n}\right)-H_{\mu }\left(Z^{N}|U^{n},S^{e}\right)\\ & \leq & H_{\mu }\left(Z^{N}|{\dot{W}}^{n}\right)-H_{\mu }\left(Z^{N}|U^{n},S^{e}\right)\\ & = & H_{\mu }\left(Z^{N}|{\dot{W}}^{n}\right)-H_{\mu }\left(Z^{N}|{\dot{W}}^{n},U^{n},S^{e}\right)\\ & \leq & H_{\mu }\left(Z^{N}|{\dot{W}}^{n}\right)-H_{\mu }\left(Z^{N}|{\dot{W}}^{n},U^{n}\right)+\log q_{e}\\ & = & I_{\mu }\left(U^{n};Z^{N}|{\dot{W}}^{n}\right)+\log q_{e}. \end{array}$$

The reliability constraint is handled exactly as in the proof of Theorem 1, except that everything should be conditioned on ${\hat{W}}^{K}$. The result of this is

(63) $$\begin{array}{ccc} I(X^{M};Y^{M}|{\hat{W}}^{K}) & \geq & H\left({\hat{U}}^{K}|{\hat{W}}^{K}\right)-K\Delta \left(\epsilon_{r}\right)-\log q_{d}\\ & \overset{▵}{=} & K\cdot R(u^{n},w^{n},q_{d},\epsilon_{r})\\ & = & M\cdot \frac{R(u^{n},w^{n},q_{d},\epsilon_{r})}{\lambda }. \end{array}$$

Regarding the security constraint, we begin with the following manipulation.

(64) $$\begin{array}{ccc} H({\hat{U}}^{K}|Z^{M},{\dot{W}}^{K}) & = & H\left({\hat{U}}^{K}|{\dot{W}}^{K}\right)-I\left({\hat{U}}^{K};Z^{M}|{\dot{W}}^{K}\right)\\ & = & H\left({\hat{U}}^{K}|{\dot{W}}^{K}\right)-H\left({\hat{U}}^{K}|{\hat{W}}^{K}\right)+H\left({\hat{U}}^{K}|{\hat{W}}^{K}\right)-I\left({\hat{U}}^{K};Z^{M}|{\dot{W}}^{K}\right)\\ & \overset{\left(a\right)}{\leq } & H\left({\hat{U}}^{K}|{\dot{W}}^{K}\right)-H\left({\hat{U}}^{K}|{\hat{W}}^{K}\right)+H\left({\hat{U}}^{K}|{\hat{W}}^{K}\right)-I\left({\hat{U}}^{K};Z^{M}|{\hat{W}}^{K}\right)+\log q_{e}\\ & = & H\left({\hat{U}}^{K}|{\dot{W}}^{K}\right)-H\left({\hat{U}}^{K}|{\hat{W}}^{K}\right)+H\left({\hat{U}}^{K}|Z^{M},{\hat{W}}^{K}\right)+\log q_{e}\\ & = & H\left({\hat{U}}^{K}|{\dot{W}}^{K}\right)-H\left({\hat{U}}^{K}|{\hat{W}}^{K}\right)+H\left({\hat{U}}^{K}|Z^{M},{\hat{W}}^{K}\right)-\\ & & H\left({\hat{U}}^{K}|Y^{M},Z^{M},S^{d},{\hat{W}}^{K}\right)+H\left({\hat{U}}^{K}|Y^{M},Z^{M},S^{d},{\hat{W}}^{K}\right)+\log q_{e}\\ & = & H\left({\hat{U}}^{K}|{\dot{W}}^{K}\right)-H\left({\hat{U}}^{K}|{\hat{W}}^{K}\right)+H\left({\hat{U}}^{K}|Z^{M},{\hat{W}}^{K}\right)-H\left({\hat{U}}^{K}|Y^{M},Z^{M},{\hat{W}}^{K}\right)+\\ & & I\left(S^{d};{\hat{U}}^{K}|Y^{M},Z^{M},{\hat{W}}^{K}\right)+H\left({\hat{U}}^{K}|Y^{M},Z^{M},S^{d},{\hat{W}}^{K}\right)+\log q_{e}\\ & \overset{\left(b\right)}{\leq } & H\left({\hat{U}}^{K}|{\dot{W}}^{K}\right)-H\left({\hat{U}}^{K}|{\hat{W}}^{K}\right)+I\left({\hat{U}}^{K};Y^{M}|Z^{M},{\hat{W}}^{K}\right)+\log q_{d}+\\ & & K\Delta \left(\epsilon_{r}\right)+\log q_{e}\\ & \leq & H\left({\hat{U}}^{K}|{\dot{W}}^{K}\right)-H\left({\hat{U}}^{K}|{\hat{W}}^{K}\right)+I\left({\hat{U}}^{K},{\hat{S}}^{e};Y^{M}|Z^{M},{\hat{W}}^{K}\right)+\log q_{d}+\\ & & K\Delta \left(\epsilon_{r}\right)+\log q_{e}\\ & \overset{\left(c\right)}{\leq } & H\left({\hat{U}}^{K}|{\dot{W}}^{K}\right)-H\left({\hat{U}}^{K}|{\hat{W}}^{K}\right)+I\left(X^{M};Y^{M}|Z^{M},{\hat{W}}^{K}\right)+\log\left(q_{e}q_{d}\right)+K\Delta \left(\epsilon_{r}\right), \end{array}$$

where in (a) we used Equation (62), in (b) we used Fano’s inequality, and in (c) we used the data processing inequality as $\left({\hat{U}}^{K},{\hat{S}}^{e}\right)\to X^{M}\to Y^{M}$ is a Markov chain (also conditioned on $\left({\hat{W}}^{K},Z^{M}\right)$). The next step is to further upper bound the term $I(X^{M};Y^{M}|Z^{M},{\hat{W}}^{K})$. This is carried out very similarly as in the proof of Theorem 1, except that everything is conditioned also on ${\hat{W}}^{K}$. We then obtain

(65) $$\begin{array}{ccc} H({\hat{U}}^{K}|Z^{M},{\dot{W}}^{K}) & \leq & H\left({\hat{U}}^{K}|{\dot{W}}^{K}\right)-H\left({\hat{U}}^{K}|{\hat{W}}^{K}\right)+M\cdot \Gamma \left[\frac{R(u^{n},w^{n},q_{d},\epsilon_{r})}{\lambda }\right]+\\ & & \log\left(q_{e}q_{d}\right)+K\Delta \left(\epsilon_{r}\right), \end{array}$$

or, equivalently,

(66) $$\begin{array}{ccc} & & H\left({\hat{U}}^{K}|{\hat{W}}^{K}\right)-M\cdot \Gamma \left[\frac{R(u^{n},w^{n},q_{d},\epsilon_{r})}{\lambda }\right]\\ & \leq & H\left({\hat{U}}^{K}|{\dot{W}}^{K}\right)-H\left({\hat{U}}^{K}|Z^{M},{\dot{W}}^{K}\right)+\log\left(q_{e}q_{d}\right)+K\Delta \left(\epsilon_{r}\right)\\ & = & I\left({\hat{U}}^{K};Z^{M}|{\dot{W}}^{K}\right)+\log\left(q_{e}q_{d}\right)+K\Delta \left(\epsilon_{r}\right)\\ & \leq & K\epsilon_{s}+\log\left(q_{e}q_{d}\right)+K\Delta \left(\epsilon_{r}\right), \end{array}$$

or

(67) $$\begin{array}{ccc} R(u^{n},w^{n},q_{e}\cdot q_{d},\epsilon_{r}) & \leq & \lambda \cdot \Gamma \left[\frac{R(u^{n},w^{n},q_{d},\epsilon_{r})}{\lambda }\right]\\ & \leq & \lambda \cdot \Gamma \left[\frac{R(u^{n},w^{n},q_{e}\cdot q_{d},\epsilon_{r})}{\lambda }\right] \end{array}$$

which is the same as

(68) $$R\left(u^{n},w^{n},q_{e}\cdot q_{d},\epsilon_{r}\right)\leq \lambda \cdot C_{s}.$$

or

(69) $$\frac{H({\hat{U}}^{K}|{\hat{W}}^{K})}{K}\leq \lambda \cdot C_{s}+\epsilon_{s}+\Delta \left(\epsilon_{r}\right)+\frac{\log(q_{e}\cdot q_{d})}{K}.$$

The proof is completed by combining the last inequality with the following inequality ([26] Equations (17)–(19), [27] Equations (55)–(57)):

(70) $$\frac{H({\hat{U}}^{K}|{\hat{W}}^{K})}{K}\geq \rho_{\mathrm{LZ}}\left(u^{n}|w^{n}\right)-\frac{\log(4A^{2})}{(1-\epsilon_{n})\log n}-\frac{A^{2}\log\left(4A^{2}\right)}{n}-\frac{1}{K},$$

where $A=[{\left(\alpha \omega \right)}^{K+1}-1]/\left[\alpha \omega -1\right]$, ω being the alphabet size of W. 

## Data Availability

Data sharing not applicable.
